# The importance of accurate developmental staging

**DOI:** 10.1093/jxb/eraa217

**Published:** 2020-06-22

**Authors:** Eric S Ober, Phil Howell, Pauline Thomelin, Allan Kouidri

**Affiliations:** NIAB, The John Bingham Laboratory, Cambridge, UK

**Keywords:** Fertility, growth stage, hybrid, meiosis, pollen development, stress, wheat

## Abstract

This article comments on:

**Fernández-Gómez J, Talle B, Tidy A, Wilson ZA.** 2020. Accurate staging of reproduction development in Cadenza wheat by non-destructive spike analysis. *Journal of Experimental Botany***71**, 3475–3484.

**Grain production depends on the successful development of reproductive structures within the nascent floral organs of cereal plants. The foundations of final yield are laid down during male and female gametogenesis, concealed within the plant. However, plant biologists, plant breeders, and agronomists often need to determine when a plant has reached particular critical stages of development. Fernández-Gómez *et al.* (2020) have developed a systematic approach to accurately determine reproductive development stages in the wheat cultivar ‘Cadenza’. This non-destructive method can accurately predict anther and pollen development through the determination of spike size and spike position within the pseudostem. This staging system can aid the development of hybrid wheat and breeding tolerance to abiotic stresses that affect fertility.**

In cereals, much of the potential grain yield is determined early in development, and hidden from view. The reproductive organs that collectively form the spike and eventually the grains begin growth and differentiation nestled within the overlapping whorls of expanding leaves that form a sheath, or pseudostem. As the true stem at the base of the spike begins to extend by expansion of successive internodes, the developing spike is raised up from the ground. Each leaf layer successively extends and unfolds away from the pseudostem as the plant grows, eventually exposing the spike which emerges from the sheath of the last ‘flag’ leaf. Flowering commences shortly after this ‘heading’, although the precise timing of developmental events depends on both the genetics of the plant and the influence of the environment. This imprecision is the bane of researchers and breeders.

Over the years, cereal scientists have devised various plant growth and development scales based on external physical appearance. A popular decimal scale was described by Zadoks (1974), and generalized into the BBCH scale (Lancashire *et al.*, 1991). An important milestone in wheat development is Zadoks growth stage 31, when the first two internodes begin to expand (Fig. 1). By this stage, the tiny developing spike has already produced the ‘terminal spikelet’, meaning that the maximum number of spikelets is now fixed. Primordial florets have formed within each spikelet (Fig. 2), many of which will eventually abort. Another critical developmental stage occurs somewhat later, when the ligule at the base of the flag leaf is visible and the rapidly developing spike has advanced into the sheath of the penultimate leaf: ‘booting’, or Zadoks growth stage 41–49 (Barber *et al.*, 2015). At booting, microsporogenesis occurs within the anthers. This early stage of pollen formation, termed S4 according to the nomenclature of Fernandez-Gomez *et al.* (2020), is especially sensitive to abiotic stresses (Parish *et al.*, 2012). For several practical reasons (detailed below), accurate ‘staging’ of growth and development is important.

**Fig. 1.**
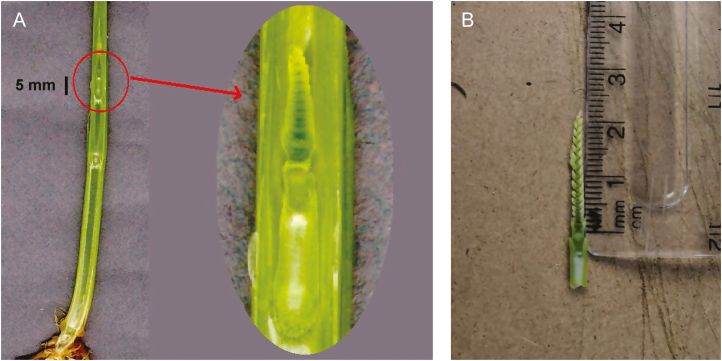
View of the developing wheat spike revealed after dissection of the pseudostem at Zadoks growth stage 31 (left). A dissected wheat spike (right) at approximately stage S2, according to Fernández-Gómez *et al.* (2020). Photo credit: Bill Clark and Phil Howell

**Fig. 2.**
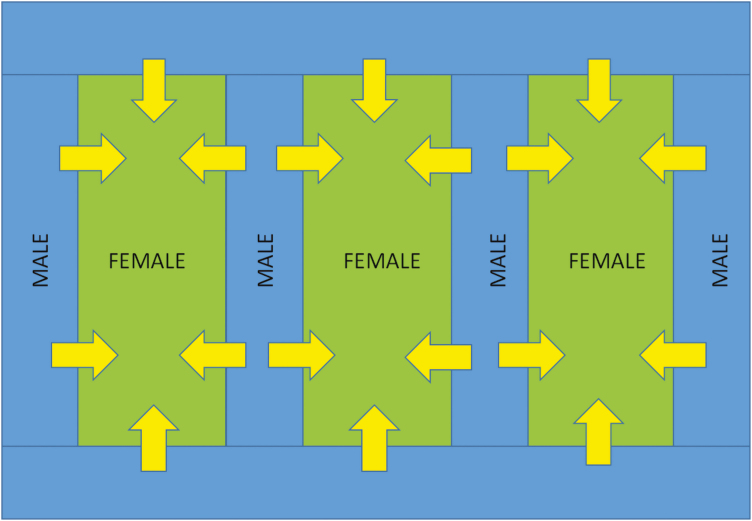
An example of a crossing block for field-scale F_1_ seed production. At the key developmental stage, female lines only (green) are treated with CHA to induce male sterility. Pollen flow (arrows) from the surrounding male line (blue) results in outcrossing and the resulting F_1_ seed harvested from the female lines is then sold to growers.

Figure 1 shows that dissection is required to accurately determine the position of the developing spike within the pseudostem and the internode length, which together define the stage. This is clearly a problem if that particular plant was needed alive at some future date, or if the plant was part of a limited genetic stock. Manual dissection is also time consuming; if many plants require assessment with limited time and resources, an alternative method is desirable that is fast, accurate, and non-destructive.

In this issue, Fernández-Gómez *et al*. report such a solution, at least for the wheat cultivar ‘Cadenza’. It has long been established that pollen development correlates with the length of the developing anther, which in turn correlates with the size and position of the developing spike (Waddington, 1983; Gómez and Wilson, 2012; Browne *et al.* 2018). Other studies established relationships between external features of the plant, such as the distance between the last two auricles (at the junctures between leaf blades and leaf sheaths), which increases as stem internodes expand and whilst the spike is developing. However, this relationship appears to be cultivar specific, perhaps due to the action of dwarfing genes and modifiers differentially affecting stem extension. Furthermore, after both the internode and spike have reached their full lengths, anther development continues and the later stages of microgametogenesis occur. Non-destructive differentiation of these final stages occurred by determining the spike position within the last leaf sheath through ‘palpation’ of the pseudostem. Noting the spike position within the leaf sheath in relation to the position of leaves and nodes, which are easily detected externally, allows this method to be adapted to any cultivar. However, spike size at key growth stages may still need to be verified for each cultivar by dissection.

Another non-destructive method of determining the size and position of the developing spike is to use X-ray micro-computed tomography (μCT) scanning (Tracy *et al.*, 2017). This was used by Fernández-Gómez *et al*. to validate their staging system until the spike was 2 cm long. Depending on the application, drawbacks to this method are the general availability of the instrumentation, scaling to high-throughput screening, and inherent difficulties for field-based measurements.

## Applications of accurate staging

Recent studies that worked out staging systems focused on varieties used to create TILLING (Targeted Induced Local Lesions in Genomes) populations: ‘Optic’ barley (Gómez and Wilson, 2012), and ‘Cadenza’ wheat (Fernández-Gómez *et al.*, 2020). The molecular physiology of genes that regulate reproductive development can be better understood when phenotypes are described in reference to the predicted stage of development at the cellular level. So too for ‘omics studies: the relative abundances of transcripts, proteins, and metabolites should be reported in reference to the more precise staging of growth and development. Comparative expression studies that make use of RNA-sequencing data should ensure that the stage of development is known for the tissues that are sampled. Several studies show the dynamic nature of expression patterns of developmental genes during anther development (Browne *et al.*, 2018; Fernández-Gómez *et al.*, 2020).

For growers and agronomists, accurately detecting the start of stem extension is essential to maximize the efficacy of crop inputs such as fertilizers, fungicides, and plant growth regulators. Application at the wrong developmental stage can reduce their effectiveness, have negative impacts on yield, or increase residue levels in harvested grain (Spink *et al.*, 2004).

As wheat naturally self-fertilizes to a high degree, field-scale F_1_ hybrid seed production depends on the ability to inhibit pollen production in some plants, but not others. Classical approaches have involved cytoplasmic male sterility and corresponding restorer genes (Gupta *et al.*, 2019) or dominant genetic male sterility (Ni *et al.*, 2017). An alternative is to apply chemical hybridizing agents (CHAs) which selectively inhibit pollen formation, resulting in male-sterile plants. These have the advantage of allowing any line to be used as male or female parents, as they do not require a maintainer line for female parent propagation or the identification and tracking of fertility restorer genes in the male parent.

The most common CHA currently licensed for use is Croisor^®^100 (sintofen), which is proprietary to ASUR Plant Breeding (Estrées-Saint-Denis, Picardy, France). Plants treated at the appropriate dose and growth stage produce non-viable pollen, thus preventing self-pollination. Other CHAs such as SQ-1 appear to induce premature programmed cell death (apoptosis) of the tapetum (Wang *et al.*, 2015), which is needed to help build the exine pollen wall and nourish the microspores (Fernández-Gómez *et al.*, 2015). Premature tapetal degeneration is also induced by tissue-specific expression of barnase enzyme in microspores of plants genetically modified with this RNase gene from *Bacillus* (Kempe *et al.*, 2014).

In F_1_ production fields, strips of the male parent are interplanted between strips of the female parent, to which CHA is applied. Ideally, the CHA will completely prevent self-pollination in the female, which is instead cross-pollinated by the neighbouring male, with F_1_ seeds harvested from the female strips only (Fig. 2). The accurate staging of crop development is fundamental to the optimal timing and dosage of CHA application, and thus to the success or failure of F_1_ seed production (Easterly *et al.*, 2019). Incomplete sterility results in significant self-pollination; this compromises the purity of the hybrid seed lot, making it unsaleable. Currently, staging involves the destructive dissection of representative female tillers in order to measure the spike length. For commercial seed production, this is relatively simple as only one female line is usually present in each production field. However, as there may be many production fields spread over a large geographical area, sown at different times and growing at different rates, each field must be staged. Furthermore, within experimental F_1_ hybrid breeding programmes, there may be many hundreds of different females in a field crossing block, albeit crudely grouped together by precocity to try and simplify CHA application.

**Fig. 3.**
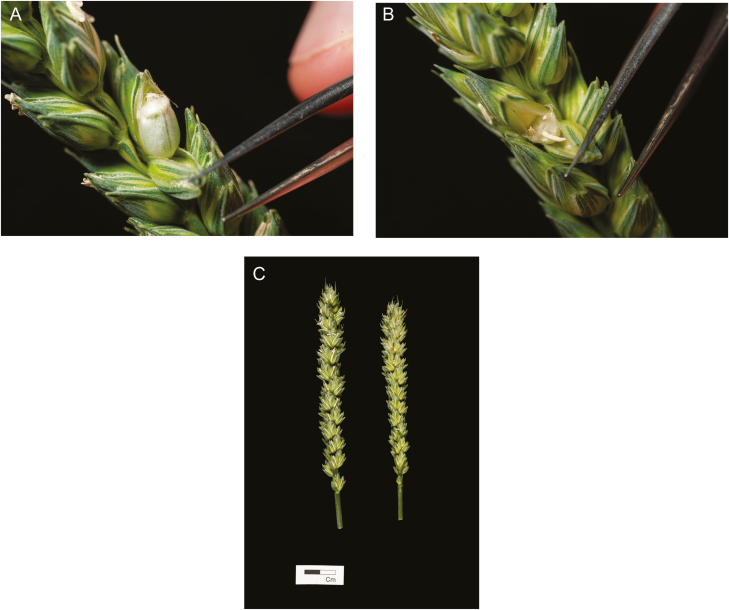
(A) A fertilized control floret and (B) a heat-stressed floret showing aborted development. (C) A control ear (left), and heat stressed ear (right). Plants (cv. Cadenza) were exposed to heat stress (35 °C day/26 °C night) for 4 d during meiosis pollen stage. Photo credit: Matt Dale

Male reproductive development is particularly sensitive to abiotic stresses, which can lead to spikelet sterility, reducing grain number and yield (Fig. 3). Worldwide, drought and heat stresses have the greatest impact on yields. Certain developmental stages are critical, and both the timing and severity of stress will determine the effects on pollen viability (Dorion *et al.* 1996; Pacini and Dolferus, 2016). As with CHA treatment, the most sensitive stages are during pollen meiosis and tapetal formation (Barber *et al.*, 2017). Stresses can also disrupt the timing of apoptosis in the tapetum, which provides substrate for the developing exine wall of the maturing pollen (De Storme and Geelen, 2013). Meiosis during microsporogenesis may occur over several days, first beginning in florets near the middle of the spike then spreading to florets at the base and the apex, and often also varying between tillers on the same plant (Barber *et al.*, 2015). Whilst male gamete formation is most sensitive to abiotic stresses, female aspects such as stigma function can also be impacted by stress (Fábián *et al.*, 2019). The breeding of stress-tolerant cultivars requires a screen that applies a reproducible level of stress at a timing that exactly corresponds to the same stage of development in each genotype. Consistency in the timing of stress application is important to differentiate genuinely stress-tolerant genotypes from those that escape stress by completing the sensitive stages of development prior to or following the stress period (Chapman *et al.*, 2012; Tricker *et al.*, 2018).

Improving the yield and yield stability of cereal crops is an important challenge for global food security. Harnessing the full potential of heterosis may boost the yields of hybrid wheat, and breeding varieties that are more resilient to abiotic stresses should help stabilize those yields. Both challenges require deep understanding of the stages of reproductive development hidden within the plant, but which need to be recognized by workers in the field using simple, fast methods that rely on exterior features. The approach outlined by Fernández-Gómez *et al.* (2020) offers much potential for improving the accuracy of staging. However, it must first be shown that their results (from ‘Cadenza’, a single, non-dwarf UK spring wheat cultivar) can be replicated across a range of winter and spring genotypes from across the globe, each selected to have phenology that matches the environment in which they are grown.

## References

[CIT0001] Barber HM, Carney J, Alghabari F, Gooding MJ 2015 Decimal growth stages for precision wheat production in changing environments? Annals of Applied Biology 166, 355–371.

[CIT0002] Barber HM, Lukac M, Simmonds J, Semenov MA, Gooding MJ 2017 Temporally and genetically discrete periods of wheat sensitivity to high temperature. Frontiers in Plant Science 8, 51. 28179910 10.3389/fpls.2017.00051 PMC5263156

[CIT0003] Browne RG, Iacuone S, Li SF, Dolferus R, Parish RW 2018 Anther morphological development and stage determination in *Triticum aestivum*. Frontiers in Plant Science 9, 228. 29527219 10.3389/fpls.2018.00228 PMC5829449

[CIT0004] Chapman SC, Chakraborty S, Dreccer MF, Howden SM 2012 Plant adaptation to climate change—opportunities and priorities in breeding. Crop and Pasture Science 63, 251–268.

[CIT0005] De Storme N, Geelen D 2013 The impact of environmental stress on male reproductive development in plants: biological processes and molecular mechanisms. Plant, Cell & Environment 37, 1–18. 10.1111/pce.12142 PMC4280902 23731015

[CIT0006] Dorion S, Lalonde S, Saini HS 1996 Induction of male sterility in wheat by meiotic-stage water deficit is preceded by a decline in invertase activity and changes in carbohydrate metabolism in anthers. Plant Physiology 111, 137–145. 12226280 10.1104/pp.111.1.137 PMC157820

[CIT0007] Easterly AC, Stroup WW, Garst N, Belamkar V, Sarazin JB, Moittié T, Ibrahim AMH, Rudd JC, Souza E, Baenziger PS 2019 Determining the efficacy of a hybridizing agent in wheat (*Triticum aestivum* L.). Scientific Reports 9, 20173. 31882883 10.1038/s41598-019-56664-9 PMC6934762

[CIT0008] Fábián A, Sáfrán E, Szabó-Eitel G, Barnabás B, Jäger K 2019 Stigma functionality and fertility are reduced by heat and drought co-stress in wheat. Frontiers in Plant Science 10, 244. 30899270 10.3389/fpls.2019.00244 PMC6417369

[CIT0009] Fernández-Gómez J, Talle B, Wilson ZA 2015 Anther and pollen development: a conserved developmental pathway. Journal of Integrative Plant Biology 57, 876–891. 26310290 10.1111/jipb.12425 PMC4794635

[CIT0010] Fernández-Gómez J, Talle B, Tidy A, Wilson ZA 2020 Accurate staging of reproduction development in Cadenza wheat by non-destructive spike analysis. Journal of Experimental Botany 71, 3475–3484. 10.1093/jxb/eraa156 PMC7307855 32255487

[CIT0011] Gómez JF, Wilson ZA 2012 Non-destructive staging of barley reproductive development for molecular analysis based upon external morphology. Journal of Experimental Botany 63, 4085–4094. 22474001 10.1093/jxb/ers092 PMC3398445

[CIT0012] Gupta PK, Balyan HS, Gahlaut V, Saripalli G, Pal B, Joshi AK 2019 Hybrid wheat: past, present and future. Theoretical and Applied Genetics 132, 2463–2483. 31321476 10.1007/s00122-019-03397-y

[CIT0013] Kempe K, Rubtsova M, Gils M 2014 Split-gene system for hybrid wheat seed production. Proceedings of the National Academy of Sciences, USA 111, 9097–9102. 10.1073/pnas.1402836111 PMC4078799 24821800

[CIT0014] Lancashire PD, Bleiholder H, Boom TVD, Langelüdeke P, Stauss R, Weber E, Witzenberger A 1991 A uniform decimal code for growth stages of crops and weeds. Annals of Applied Biology 119, 561–601.

[CIT0015] Ni F, Qi J, Hao Q, et al 2017 Wheat Ms2 encodes for an orphan protein that confers male sterility in grass species. Nature Communications 8, 15121. 10.1038/ncomms15121 PMC5414350 28452349

[CIT0016] Pacini E, Dolferus R 2016 The trials and tribulations of the plant male gametophyte—understanding reproductive stage stress tolerance. In: Shanker AK, Shanker C, eds. Abiotic and biotic stress in plants—recent advances and future perspectives. IntechOpen, London, UK. doi:10.5772/61671.

[CIT0017] Parish RW, Phan HA, Iacuone S, Li SF 2012 Tapetal development and abiotic stress: a centre of vulnerability. Functional Plant Biology 39, 553–559. 32480807 10.1071/FP12090

[CIT0018] Spink J, Berry PM, Wade AP, White EM 2004 Effects of timing and dose on residues of chlormequat in wheat, barley and oats. HGCA Project Report No 334.

[CIT0019] Tracy SR, Gómez JF, Sturrock CJ, Wilson ZA, Ferguson AC 2017 Non-destructive determination of floral staging in cereals using X-ray micro computed tomography (µCT). Plant Methods 13, 9. 28261319 10.1186/s13007-017-0162-x PMC5331626

[CIT0020] Tricker PJ, ElHabti A, Schmidt J, Fleury D 2018 The physiological and genetic basis of combined drought and heat tolerance in wheat. Journal of Experimental Botany 69, 3195–3210. 29562265 10.1093/jxb/ery081

[CIT0021] Waddington SR, Cartwright PM, Wall PC 1983 A quantitative scale of spike initial and pistil development in barley and wheat. Annals of Botany 51, 119–130.

[CIT0022] Wang S, Zhang G, Song Q, Zhang Y, Li Z, Guo J, Niu N, Ma S, Wang J 2015 Abnormal development of tapetum and microspores induced by chemical hybridization agent SQ-1 in wheat. PLoS One 10, e0119557. 25803723 10.1371/journal.pone.0119557 PMC4372346

[CIT0023] Zadoks JC, Chang TT, Konzak CF 1974 A decimal code for the growth stages of cereals. Weed Research 44, 415–421.

